# Research on Direction of Arrival Estimation Based on Self-Contained MEMS Vector Hydrophone

**DOI:** 10.3390/mi13020236

**Published:** 2022-01-30

**Authors:** Shan Zhu, Guojun Zhang, Daiyue Wu, Xiaoqi Liang, Yifan Zhang, Ting Lv, Yan Liu, Peng Chen, Wendong Zhang

**Affiliations:** State Key Laboratory of Dynamic Measurement Technology, North University of China, Taiyuan 030051, China; s2006026@st.nuc.edu.cn (D.W.); s1906143@st.nuc.edu.cn (X.L.); s2006042@st.nuc.edu.cn (Y.Z.); s1906036@st.nuc.edu.cn (T.L.); b200612@st.nuc.edu.cn (Y.L.); s1906084@st.nuc.edu.cn (P.C.); wdzhang@nuc.edu.cn (W.Z.)

**Keywords:** MEMS, vector hydrophone, self-contained, DOA, beamforming

## Abstract

A self-contained MEMS vector hydrophone with a scalar–vector integrated design is proposed in this paper. Compared with traditional MEMS vector hydrophones, this design solves the problem of ambiguity in the port and starboard during orientation, and also realizes the self-contained storage of acoustic signals. First, the sensor principle and structural design of the self-contained MEMS hydrophone are introduced, and then the principle of the combined beamforming algorithm is given. In addition to this, the amplitude and phase calibration method based on the self-contained MEMS vector hydrophone is proposed. Then, the sensitivity and phase calibrations of the sensor are carried out in the standing wave tube. The sensitivity of the vector channel is −182.7 dB (0 dB@1 V/μPa) and the sensitivity of the scalar channel is −181.8 dB (0 dB@1 V/μPa). Finally, an outdoor water experiment was carried out. The experimental results show that the self-contained MEMS vector hydrophone can accurately pick up and record underwater acoustics information. It realizes the precise orientation of the target by combining beamforming algorithms. The direction of arrival (DOA) error is within 5° under the outdoor experimental conditions with an SNR of 13.67 dB.

## 1. Introduction

The vector hydrophone has the advantage of being able to estimate the azimuth of the target by a single one, so it has been widely used in underwater passive detection in recent years. In 1956, Leslie et al. first fabricated a hydrophone that measured the flow velocity of water by installing a velocity pickup inside a rigid spherical housing [1]. Based on this research, TB Gabrielson et al. developed a hydrophone with the same principle, which consists of a glass micro-balloon and epoxy composite cast around a small commercial geophone. In conjunction with a pressure hydrophone, acoustic intensity can be measured by this sensor [2]. In 1998, the first piezoelectric, flexural-disk was designed that was inherently a buoyant underwater accelerometer [3]. By evolving microelectromechanical (MEMS) technology, in 1999, a MEMS hydrophone was presented by Boston University that could detect the acoustic field variations by detecting a reflected laser beam [4]. In 2007, Xue et al. introduced a novel microelectromechanical systems (MEMS) bionic vector hydrophone, with a receiving sensitivity of −197.7 dB (0 dB@1 V/μPa). It integrates MEMS technology, bionics and piezoresistive effect, and has the advantages of excellent low-frequency sensitivity and miniaturization [5,6,7]. Following this idea, researchers have developed several modified sensors, which have achieved a higher sensitivity and wider frequency bands [8,9,10,11].

With the widespread application of vector hydrophones, the signal processing of vector hydrophones has become an important research direction in the field of underwater acoustics. The research groups of Arye Nehorai at Yale University in the United States have made great contributions. In 1992, A Nehorai et al. introduced two simple algorithms for estimating the source DOA with a vector hydrophone [12]. In 1996, B Hochwald et al. studied spatial resolution capability based on the acoustic vector array and deduced the maximum number of sound sources that the vector array can distinguish [13]. A novel ESPRIT-based closed-form source localization algorithm applicable to arbitrarily spaced three-dimensional arrays of vector hydrophones was proposed by KT Wong et al. in 1997 [14,15]. M Hawkes et al. used arrays of acoustic vector sensors for beamforming and Capon direction of arrival (DOA) estimation, and derived an expression for the Cramer–Rao bound (CRB) on the DOA parameters of a single source [16]. In 2000, J Hui et al. proposed a signal processing algorithm combining pressure and particle velocity information, which has better processing results than traditional underwater acoustic algorithms that just utilize pressure information [17]. In 2006, ZX Yao et al. proposed a histogram statistical azimuth estimation approach based on single vector hydrophone and carried out sea trials. The results of a sea trial show the validity of suppressing the coherent line-spectra interference [18]. Since 2013, researchers have successively conducted research on vector underwater acoustic signal processing of different types of sensors applied in different scenarios [19,20,21]. The research of K Kinu et al. demonstrated the application of underwater vector sensors in source localization and target tracking, and verified the good performance of source DOA based on the conventional beamforming technique through experiments [22].

At present, the research on the signal processing algorithm of the ciliated MEMS vector hydrophone is still in its infancy. Shang et al. studied the orientation algorithm of the MEMS vector hydrophone and gave experimental results [23]. However, they used the NI PXIe-6358 data acquisition card for data acquisition, which has drawbacks. The noise introduced by the cable of more than ten meters will affect the orientation accuracy. A mixed near-field and far-field source localization algorithm based on a MEMS vector hydrophone array was studied by Shang et al. Unfortunately, they only gave the simulation analysis of the algorithm, and did not carry out an experimental verification [24]. On the basis of the research of Shang et al., we design a self-contained MEMS vector hydrophone in this paper. In addition to solving the problem of starboard and starboard ambiguity in orientation, it also realizes the self-contained storage of acoustic signals. During the experiment, the hydrophone was no longer dependent on the shore system, eliminating the noise introduced by the long cable. At the same time, the fidelity of the data is improved, and it is convenient for carrying with the underwater platform. This is more in line with the needs of MEMS vector hydrophone engineering applications. A target orientation experiment was carried out in a standing wave tube and outdoor water, and the performance of the self-contained MEMS vector hydrophone was verified by the combined beamforming algorithm.

## 2. Self-Contained MEMS Vector Hydrophone

### 2.1. Sensitive Principle of the Sensor

**Figure 1:**
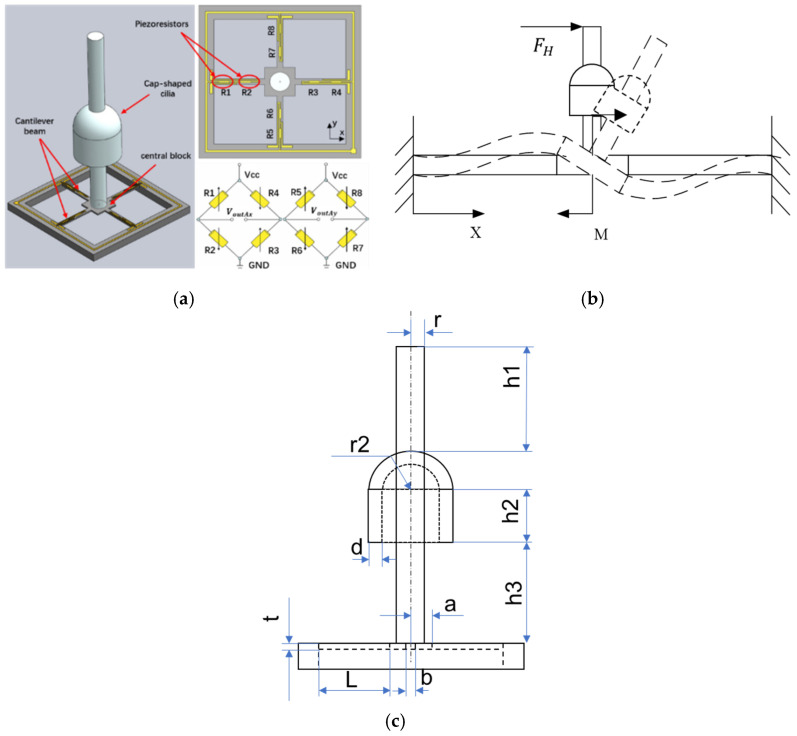
(**a**) Sensitive structure of vector channel. (**b**) Schematic diagram of stress on beam. (**c**) Geometric parameter schematic of vector channel.

**Figure 2:**
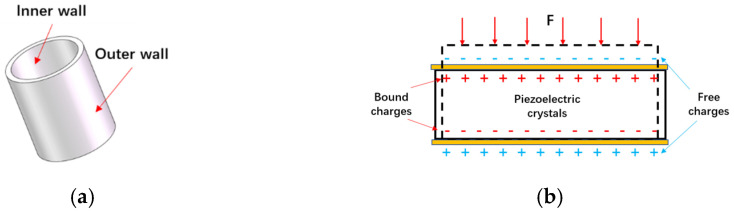
(**a**) Sensitive structure of sound pressure channel; (**b**) principle diagram of piezoelectric effect.

The self-contained MEMS vector hydrophone proposed in this paper is a 2D vector underwater acoustic sensor. It adopts a scalar–vector integrated design, including two vector channels and an acoustic pressure channel, which can simultaneously pick up the acoustic pressure and vibration velocity information of the sound field. The cap-shaped cilia design allows the sensor to have a higher sensitivity, and its production process is given in the paper by Zhu et al. [11]. The sensitive structure of the sensor vector channel (ciliary cross beam, Wheatstone bridge and piezoresistor position) is shown in Figure 1, and the main parameters of the vector microstructure are shown in Table 1. Its manufacturing process is introduced in the study of Xue et al. [6,7]. When the cilia oscillate under the action of acoustic waves, the cantilever beam will be deformed under force. According to the piezoresistive effect, the resistance value of the piezoresistor attached to the cantilever beam also changes. Then, two Wheatstone bridges are used to convert the variation of the piezoresistor into a voltage variation as the output. The acoustic pressure channel-sensitive structure (piezoelectric ceramic tube) is shown in Figure 2. When acoustic pressure acts on the ceramic tube, the inner and outer walls of the ceramic tube will generate charges with opposite polarities, thereby completing the conversion of acoustic pressure signals to electrical signals.

The mechanical analysis of the cantilever beam is carried out below. The variation of the piezoresistor is proportional to the stress on the beam. The force of the cantilever beam is shown in Figure 1b. According to previous studies and the principles of material mechanics, the stress at any point on the cantilever beam σ(x) can be expressed as follows [8]:

(1) $$\begin{array}{cc} \sigma (x) & =\pm \frac{L^{2}+3aL-3x(a+L)}{\frac{2}{3}bt^{2}(L^{2}+3aL+3a^{2})}M\pm \frac{F_{H}}{bt}=\pm \frac{L^{2}+3aL-3x(a+L)}{\frac{2}{3}bt^{2}(L^{2}+3aL+3a^{2})}F_{H}H^{\prime }\pm \frac{PS}{bt}\\ & =\pm 2P(\frac{L^{2}+3aL-3x(a+L)}{\frac{2}{3}bt^{2}(L^{2}+3aL+3a^{2})}H^{\prime }\pm \frac{1}{bt})\;(rh_{1}+r_{2}{}^{2}+r_{2}h_{2}+rh_{3}) \end{array}$$

Here, M is the moment at the central junction of cantilever beams; $F_{H}$ is the force acting on the cilia in the horizontal direction; *S* is the area receiving the sound signal; *P* is the external sound pressure; $H^{\prime }$ is the height of the center of gravity of the cilia structure; and other parameters are shown in Figure 1c.

### 2.2. Encapsulation Design of Self-Contained MEMS Vector Hydrophone

**Figure 3:**
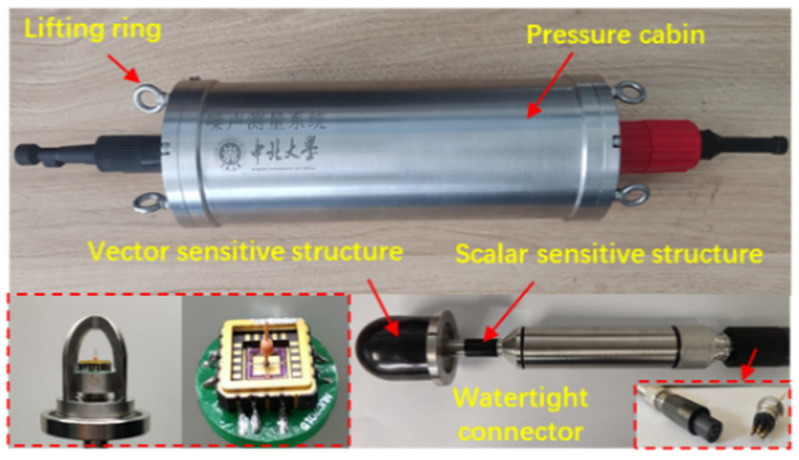
Overall structure diagram.

**Figure 4:**
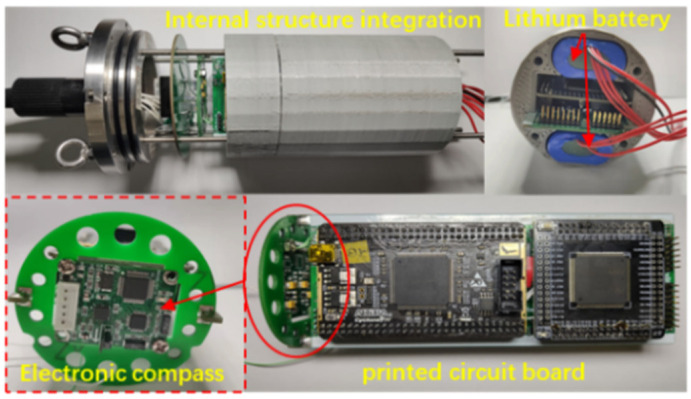
Internal structure integration of electronic cabin.

The self-contained MEMS vector hydrophone has ideal low-frequency performance and attitude calibration capabilities. Its internal data storage unit can record the acoustic signals picked up by the sensitive structure of the sensor. In order to accurately record underwater sounds, the sampling frequency and sampling depth of the recorder are set reasonably, and all channels are equipped with high-precision clocks to achieve synchronous sampling between channels. The electronic cabin is powered by a lithium battery pack, and uses a 64G SD Card to ensure that the system can work continuously under water for more than 48 h. The sensitive structure of the sensor is encapsulated in a support frame made of stainless steel in order to protect the microstructure from damage. A cap made of polyurethane material is installed on the outside of the support frame for sound transmission. The overall structure is shown in Figure 3. A pressure-resistant electronic cabin with a hydrostatic pressure resistance of 4.5 MPa was designed, in which the attitude sensor, circuit board and lithium battery were placed, and, in said design, the sensor probe is rigidly connected to the electronic cabin through a watertight connector. The internal structure integration is shown in Figure 4. The north direction of the attitude sensor coincides with the positive direction of the X axis of the sensor, and the real-time attitude data of the sensor are stored synchronously with the acoustic signal to realize the attitude calibration of the hydrophone.

### 2.3. Target Recognition Characteristics of Self-Contained MEMS Vector Hydrophone

**Figure 5:**
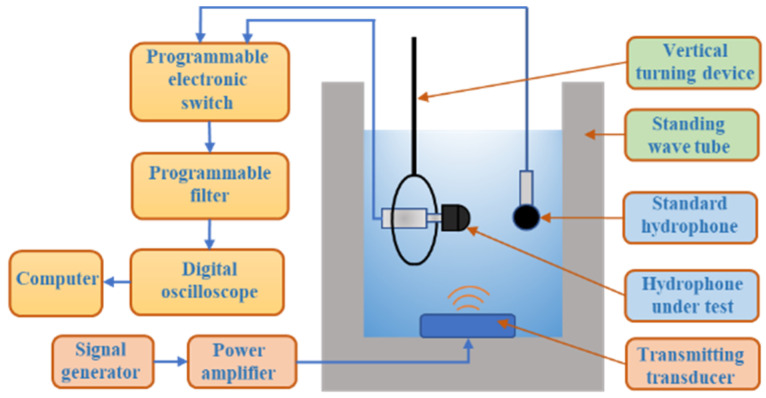
Schematic diagram of hydrophone calibration system.

A standard low-frequency hydrophone calibration system is used to calibrate the self-contained MEMS vector hydrophone in a standing wave tube. The calibration process is shown in Figure 5. A sounding transducer is installed at the bottom of the standing wave tube, the vector hydrophone is fixed on the gyro device during calibration, and the working state of the calibration system is controlled by a computer. The sensitivity of the sensor is calibrated by the comparative calibration method [25] and the sensitivity calculation formula is shown as Equation (2) [26]:

(2) $$M_{x}=20\mathrm{lg}\left(\frac{U_{x}}{U_{0}}\frac{sinkd}{coskd_{0}}\right)+M_{0}$$

Here, $M_{x}$ is the sound pressure sensitivity of the vector hydrophone; $M_{0}$ is the sound pressure sensitivity of the standard hydrophone ($M_{0}$ = −170 dB); $U_{x}$ is the output voltage of the vector hydrophone; $U_{0}$ is the output voltage of the standard hydrophone; $d_{0}$ is the underwater depth of the standard hydrophone; d is the underwater depth of the vector hydrophone; k is the wave number, k=2πf/c; f is the frequency; and c is the speed of sound.

During the directivity test, the gyro device rotates in steps of 1°, and the system records the output voltage of the hydrophone at each angle. According to the directivity calculation formula, the directivity of the hydrophone is finally drawn on the polar coordinate system. The directivity calculation formula is shown in Equation (3) [26]:

(3) $$L=20\mathrm{lg}\left(\frac{U_{\theta }}{U_{max}}\right)$$

wherein: θ is the angle of current rotation; $U_{\theta }$ is the real-time output voltage value of each angle; and $U_{max}$ is the maximum output voltage value of the hydrophone.

**Figure 6:**
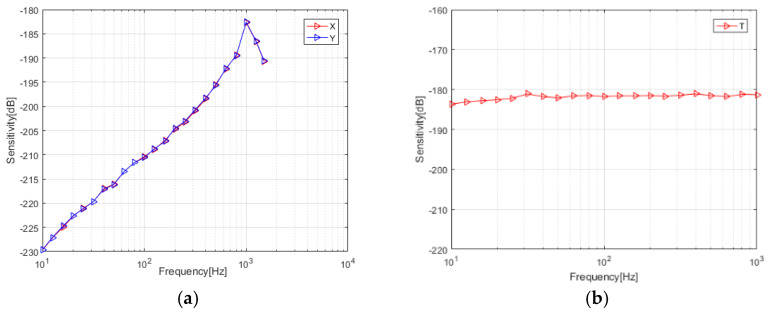
Sensitivity curve of sensor. (**a**) Vector channel; (**b**) scalar channel.

**Figure 7:**
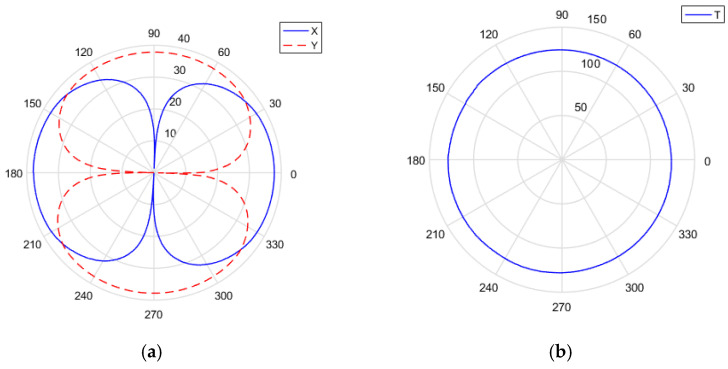
Directivity diagrams of sensor. (**a**) Vector channel; (**b**) scalar channel.

The sensitivity curve of the sensor is shown in Figure 6a,b, the vector channel sensitivity is −182.7 dB @1 kHz (0 dB@1 V/μPa), and the scalar channel sensitivity is −181.8 dB (0 dB@1 V/μPa). The test results show that the sensor has a good low-frequency performance, and the vector channel sensitivity curve increases by 2 dB every 1/3 octave, which is in line with the characteristics of an acoustic pressure gradient hydrophone. The directivity diagram measured at 315 Hz is shown in Figure 7a,b. The pit depth of the “8”-shaped directivity diagram of the two vector channels is more than 30dB. The directivity and consistence of the hydrophone is good, and the scalar channel has a relatively complete omnidirectional directivity.

## 3. Principles of Azimuth Estimation Algorithm

### 3.1. Principles of Beamforming Algorithm

The standard-vector integrated (composite) MEMS vector hydrophone designed in this paper contains one sound pressure channel and two vibration velocity channels. Combining them can yield more sound field information. Therefore, using the combined beamforming algorithm to jointly process the sound pressure and vibration velocity information can realize the DOA estimation of a single vector hydrophone, while avoiding the problem of port and starboard ambiguity.

The sound pressure is non-directional and can be expressed as Equation (4).

(4) p(t)=x(t)

Let the sound field satisfy the plane wave Ohm’s law, as shown in Equation (5).

(5) $$\frac{p\left(t\right)}{v\left(t\right)}=\rho c$$

Then, the vibration speed v(t) can be expressed as:

(6) $$v\left(t\right)=\frac{1}{\rho c}x\left(t\right)$$

From the sound wave plane projection relationship, we obtain:

(7) $$\{\begin{array}{c} v_{x}\left(t\right)=v\left(t\right)cos\theta \\ v_{y}\left(t\right)=v\left(t\right)sin\theta \end{array}$$

Incorporating Equation (6) into Equation (7), we can obtain:

(8) $$\{\begin{array}{c} v_{x}\left(t\right)=\frac{1}{\rho c}x\left(t\right)cos\theta \\ v_{y}\left(t\right)=\frac{1}{\rho c}x\left(t\right)sin\theta \end{array}$$

When discussing signal processing problems, for the sake of a brief description, the acoustic impedance ρc in Equation (8) is generally omitted and set to 1 (it cannot be omitted when discussing acoustic problems and physical dimensions are involved). So, the directivity of the composite MEMS vector hydrophone in the horizontal direction can be expressed as Equation (9).

(9) $$\{\begin{array}{c} p\left(t\right)=x\left(t\right)\\ v_{x}\left(t\right)=x\left(t\right)cos\theta \\ v_{y}\left(t\right)=x\left(t\right)sin\theta \end{array}$$

In Equation (9), x(t) is the acoustic pressure waveform and θ is the horizontal azimuth angle. $v_{x}$ and $v_{y}$ represent the directivity of the dipoles orthogonal to each other.

**Figure 8:**
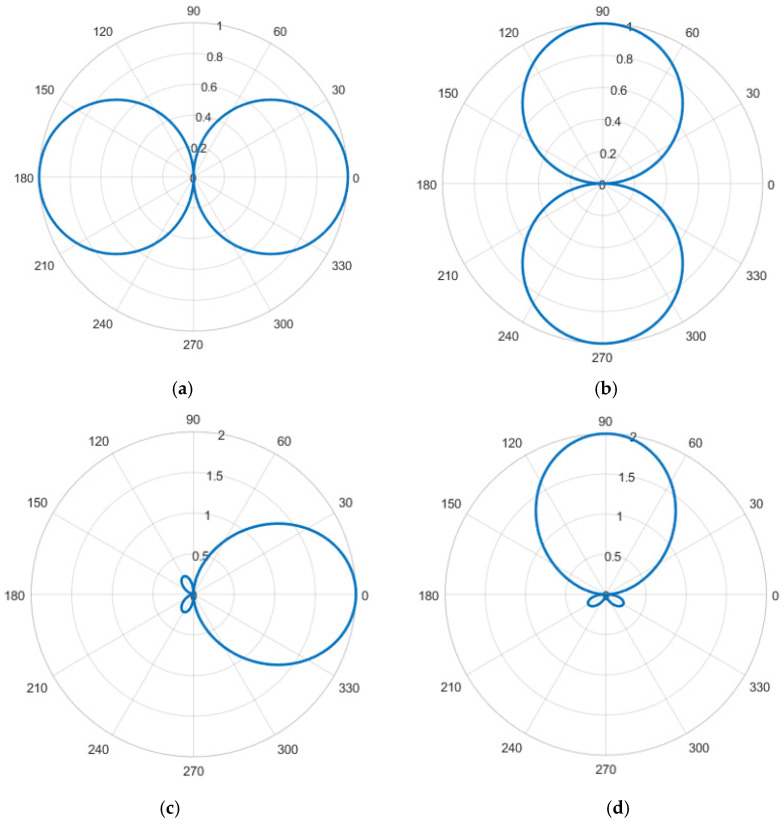
Directivity map. (**a**,**b**) $v_{c}\left(t\right)$; (**c**,**d**) $\left[p\left(t\right)+v_{c}\left(t\right)\right]v_{c}\left(t\right)$.

After combining the two orthogonal components $v_{x}$ and $v_{y}$, the combined vibration velocity $v_{c}$ and $v_{s}$ are shown in Equation (10).

(10) $$\{\begin{array}{c} v_{c}\left(t\right)=v_{x}\left(t\right)cos\psi +v_{y}\left(t\right)sin\psi \\ v_{s}\left(t\right)=-v_{x}\left(t\right)sin\psi +v_{y}\left(t\right)cos\psi \end{array}$$

wherein ψ is the guide azimuth (0–360°). Incorporating Equation (9) into Equation (10), we can obtain:

(11) $$\{\begin{array}{c} v_{c}\left(t\right)=x\left(t\right)cos\left(\theta -\psi \right)\\ v_{s}\left(t\right)=x\left(t\right)sin\left(\theta -\psi \right) \end{array}$$

wherein the combined vibration velocity $v_{c}$ and $v_{s}$ still have orthogonal dipole directivity, and the directivity is shown in Figure 8a,b. The guide azimuth angle ψ is the main maximum direction of $v_{c}$. When ψ is changed in all directions (0–360°) through electronic rotation, and θ=ψ, the direction of the maximum amplitude is the direction of the target.

In order to achieve unambiguous orientation to the target, a new weighted combination of acoustic pressure and vibration velocity is used to form a new combination of vibration velocity with a single directivity, as shown in Equation (12).

(12) $$\left[p\left(t\right)+v_{c}\left(t\right)\right]v_{c}\left(t\right)=x^{2}\left(t\right)cos\left(\theta -\psi \right)\left[1+cos\left(\theta -\psi \right)\right]$$

The corresponding directivity can be expressed as Equation (13).

(13) $$R\left(\theta \right)=cos^{2}\frac{\theta -\psi }{2}\;cos\left(\theta -\psi \right)$$

The directivity map of the combined vibration velocity is “tadpole-shaped”, showing a single directivity. The directivity diagram is shown in Figure 8c,d.

### 3.2. Principle of Amplitude and Phase Calibration

**Figure 9:**
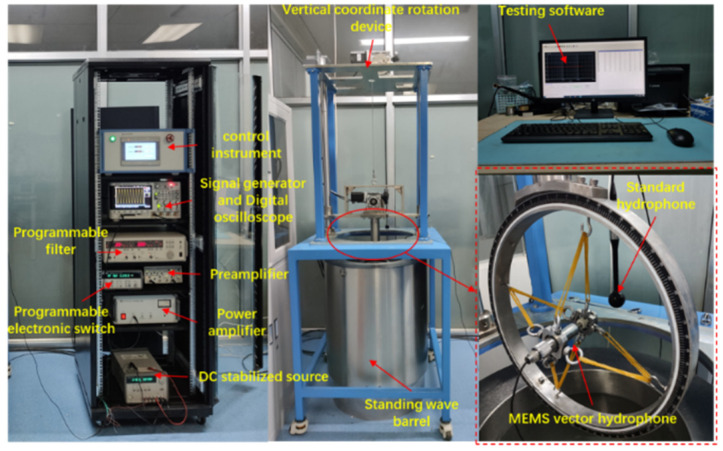
Calibration site in standing wave tube.

**Figure 10:**
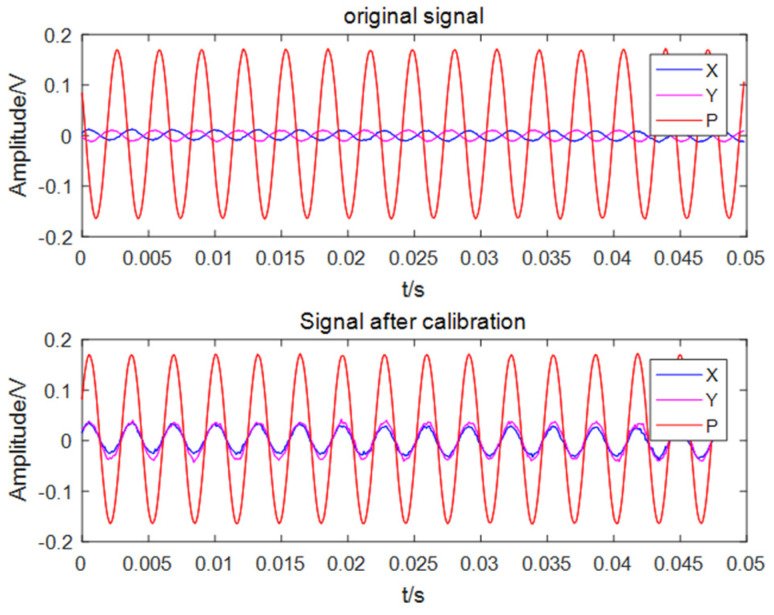
Phase-calibrated signal and original signal.

The composite MEMS vector hydrophone can simultaneously and independently collect the acoustic pressure and particle velocity information at the same point in the sound field. It has three channels, which output the projection components of the acoustic pressure and particle velocity vectors along the x and y axes in the Cartesian coordinate system of the observation point. The acoustic pressure of the coherent source and the particle velocity are completely correlated in the far-field region, and there is no phase difference between them [27]. However, the scalar and vector channels of the composite MEMS vector hydrophone are made of different sensitive units, so there may be inconsistencies in the signal phase and amplitude between the scalar and vector channels. When the combined algorithm is used to DOA estimation of a single vector hydrophone, the phase difference between the scalar and vector channels will change the eigenvectors of the receiving matrix, which will cause errors in the orientation results. In order to eliminate the orientation error caused by the phase difference between the channels, the sensor needs to calibrate its amplitude and phase after production. The calibration site is shown in Figure 9. The calibration process is completed in the standing wave tube. First, the 1/3-octave sound signal is sequentially transmitted through the transmitting transducer at the bottom of the standing wave tube. Then, the standard hydrophone is used as the reference channel, and each signal received by the hydrophone is compared with the signal of the reference channel. Finally, we record the signal amplitude and the phase difference with the reference channel of each measured channel at different frequency points for later data processing. The signal amplitude and phase calibration results of each channel at 315 Hz are shown in Figure 10.

**Figure 11:**
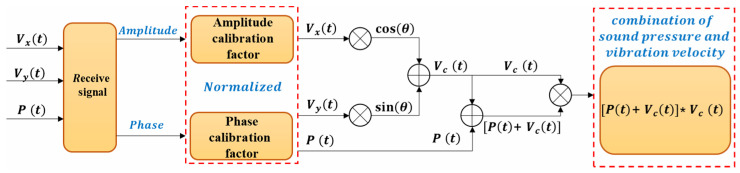
Principle block diagram of beamforming algorithm.

In practical applications, it is necessary to calibrate the amplitude and phase of each channel, and then use the acoustic pressure-vibration velocity combined beamforming algorithm to DOA estimation. The principle of the beamforming algorithm after the amplitude and phase are corrected is shown in Figure 11.

The three signals of the calibrated hydrophone are shown in Equation (14).

(14) $$\{\begin{array}{c} p\left(t\right)=A_{1}\cdot x\left(t+\tau_{1}\right)\\ v_{x}\left(t\right)=A_{2}\cdot x\left(t+\tau_{2}\right)cos\theta \\ v_{y}\left(t\right)=A_{3}\cdot x\left(t+\tau_{3}\right)sin\theta \end{array}$$

wherein $A_{1},A_{2},A_{3}$ represent the sensitivity compensation coefficient and $\tau_{1},\tau_{2},\tau_{3}$ represent the phase difference between the three signals.

The combined vibration velocity $v_{c}$ and $v_{s}$ are shown in Equation (15).

(15) $$\{\begin{array}{c} v_{c}\left(t\right)=A_{2}\cdot x\left(t+\tau_{2}\right)cos\theta cos\psi +A_{3}\cdot x\left(t+\tau_{3}\right)sin\theta sin\psi \\ v_{s}\left(t\right)=-A_{2}\cdot x\left(t+\tau_{2}\right)cos\theta sin\psi +A_{3}\cdot x\left(t+\tau_{3}\right)sin\theta cos\psi \end{array}$$

The combination of sound pressure and vibration velocity can be expressed as Equation (16).

(16) $$\begin{array}{c} \left[p\left(t\right)+v_{c}\left(t\right)\right]v_{c}\left(t\right)=\left[A_{1}x\left(t+\tau_{1}\right)+A_{2}x\left(t+\tau_{2}\right)cos\theta cos\psi +A_{3}x\left(t+\tau_{3}\right)sin\theta sin\psi \right]\\ \cdot \left[A_{2}x\left(t+\tau_{2}\right)cos\theta cos\psi +A_{3}x\left(t+\tau_{3}\right)sin\theta sin\psi \right] \end{array}$$

## 4. Experiment

### 4.1. Indoor Experiment

**Figure 12:**
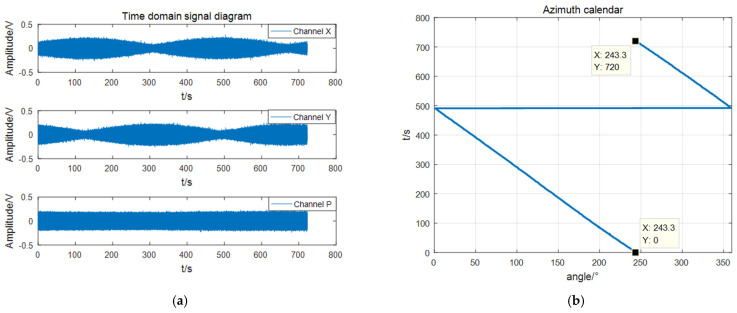
Signal processing results: (**a**) time domain diagram of three-channel signals; (**b**) azimuth history diagram of the measured target.

In order to verify the directional algorithm proposed in Section 3, the DOA estimation performance of the self-contained MEMS vector hydrophone was tested in a standing wave tube. The test site is shown in Figure 9. The test process is detailed. First, the MEMS vector hydrophone probe was fixed on the automatic gyroscope. Then, the computer caused the gyroscope to rotate clockwise at a speed of 30°/min. The rotation interval was [180°, −180°] of the gyroscope coordinates, which corresponded to [243°, 0°] and [360°, 243°] of the hydrophone coordinates. At the same time, the sound source at the bottom of the standing wave tube emitted a 250 Hz continuous sine signal (SNR = 23.34 dB). The experimental data were collected and stored through a self-contained collection and storage system, and the system sampling rate was 40 ksps. After the experiment, the combined algorithm was used to DOA estimation in MATLAB. In the calculation, the sliding window step distance was selected for ΔT=1 s, and the total analysis time was 720 s. The result of the data processing is shown in Figure 12. The signal time domain graph shows that the amplitudes of the X and Y signals were orthogonal to each other in the process of rotation. The azimuth angle changed from 243.3° to 0°, and from 360° to 243.3°. The interval time length was 720 s, the angle changed by 360°, which was the same as the treatment set by the gyroscope, and the trajectory was smooth without jitter. The test results showed that the acoustic pressure-vibration velocity combined beamforming algorithm proposed in this paper can avoid the problem of ambiguity on the port and starboard, and can realize accurate positioning and tracking of the sound source. The indoor measurement angle error is within 1°.

### 4.2. Outdoor Water Experiment

**Figure 13:**
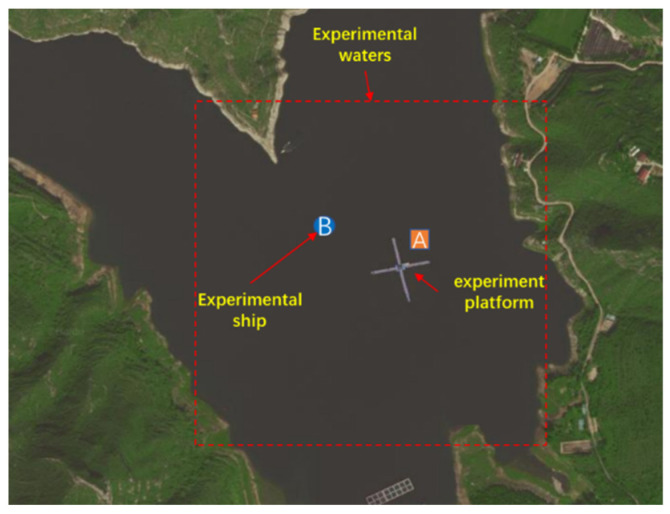
Outdoor experimental water area map.

In order to further verify the DOA estimation capability of the self-contained MEMS vector hydrophone, a reservoir experiment was carried out in open outdoor water. The experimental site was selected at the Fenhe Reservoir in the northwest of Taiyuan City. The open area of the experimental water area is 1 km^2^, and the average water depth is 30 m, which is a relatively good experimental environment. The experimental waters are shown in Figure 13. The experiment was carried out on a cross-shaped floating platform built in the center of the reservoir. The experimental equipment included a mobile power supply, the self-contained MEMS vector hydrophone, a signal generator, a fish lip transducer, a portable GPS locator and a computer.

#### 4.2.1. Fixed Target Experiment

**Figure 14:**
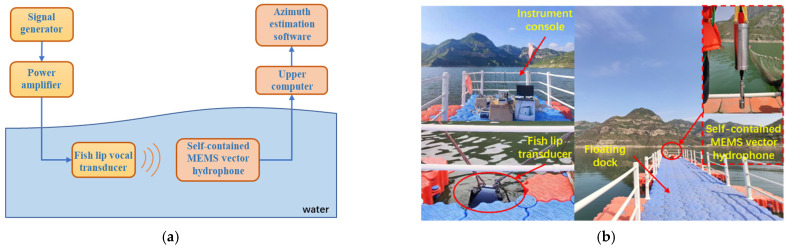
(**a**) Schematic diagram of experimental process; (**b**) diagram of experimental site.

**Figure 15:**
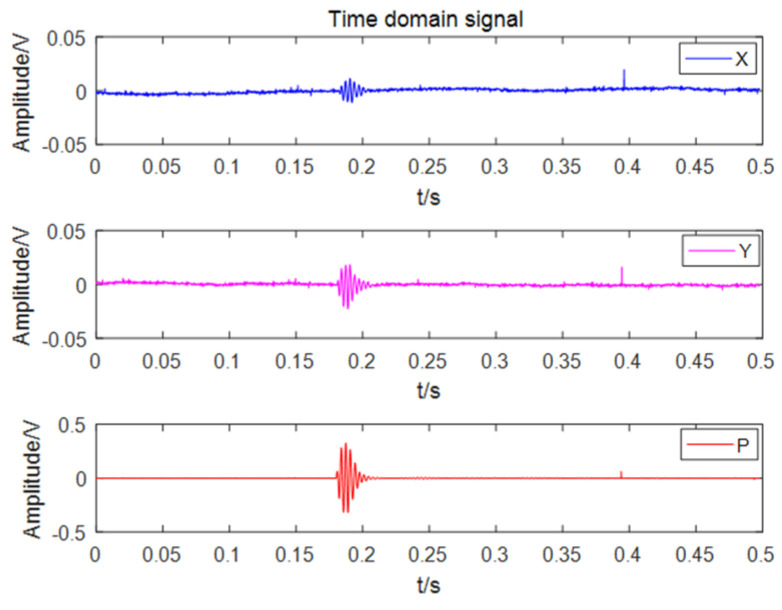
Time domain diagram of pulse signal.

**Figure 16:**
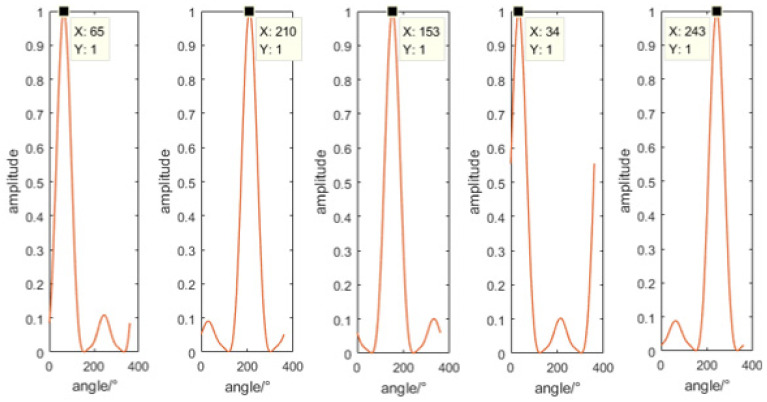
Direction of arrival estimation results.

The fixed sound source transmitting and receiving experiment was carried out on the cross-shaped floating platform A, and the experiment was divided into five groups. The schematic diagram of the experimental process is shown in Figure 14a. The transmitting system is composed of a signal generator, a power amplifier and a fish lip transducer, and the receiving system is composed of a self-contained MEMS vector hydrophone and a computer. Before the experiment, the self-contained MEMS vector hydrophone was programmed by the host computer to start working after a delay of 20 min. Then, the self-contained MEMS vector hydrophone and the fish lip transducer were lowered to a depth of 15 m underwater in five sets of different positions and angles. The experimental site is shown in Figure 14b. The audible signal selected a 315 Hz pulse signal, and emitted 3 cycles every 1 s (SNR = 13.67 dB). The position information of the sound source and the hydrophone was recorded by GPS, and the angle information was calculated using the GPS position angle converter. The angles of the five sets of position sound sources relative to the hydrophone are [62.21°, 211.86°, 156.67°, 34.49°, 247.23°]. The three pulse signals received by the hydrophone are shown in Figure 15. The combined algorithm is used to DOA estimation, and the calculated beam pattern is shown in Figure 16. The experimental results show that the beam pattern of the combined algorithm does not have the problem of ambiguity on the port and starboard, and achieves a unique orientation with a maximum orientation error of 4.23°.

#### 4.2.2. Moving Target Experiment

**Figure 17:**
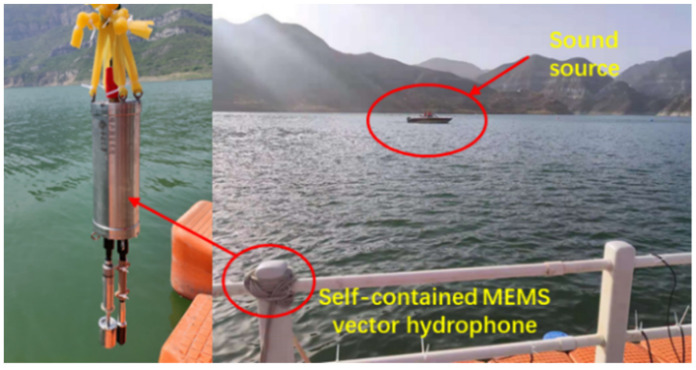
Experimental field of ship noise acquisition.

**Figure 18:**
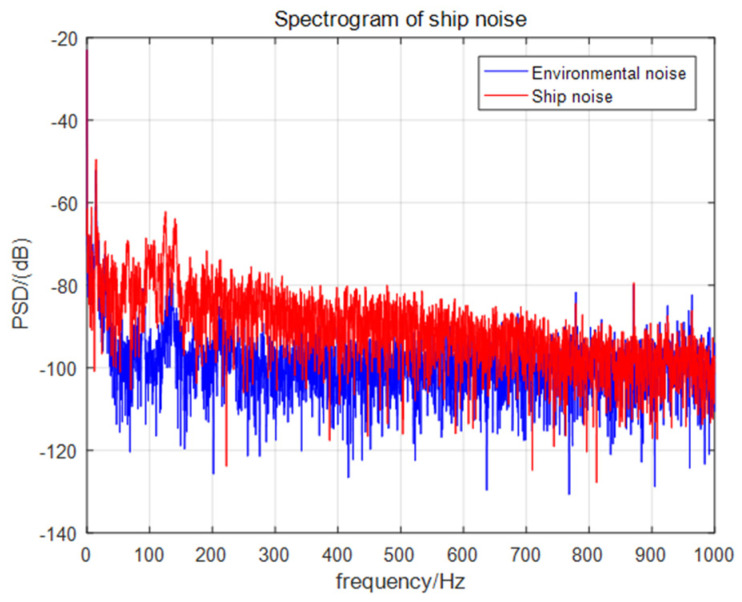
Power spectral density of ship noise.

**Figure 19:**
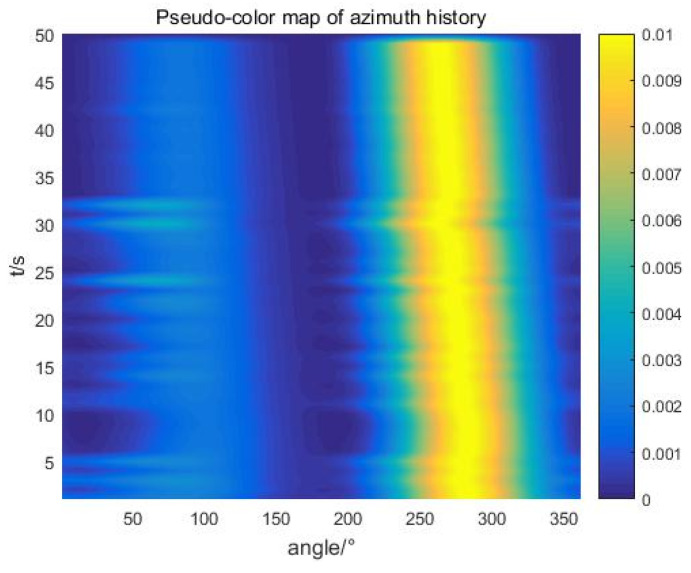
Waterfall diagram of ship target.

A mobile target test was carried out in the test water area, taking the ship as a single target sound source, and the self-contained MEMS vector hydrophone was lowered from the floating platform to a depth of 15 m underwater. The experimental site is shown in Figure 17. The ship orbited clockwise around the floating dock and used GPS to record the track information. The noise of the speedboat was collected and stored by the self-contained MEMS vector hydrophone. After the experiment, the data were read out for trajectory analysis. The ship noise power spectrum is shown in Figure 18. The power spectral density results show that the frequency band of broadband noise is mainly concentrated in the low frequency range of 50–800 Hz and contains some line spectrum information. The main line spectrum characteristics are 15 Hz, 125 Hz, 140 Hz, 779 Hz and 871 Hz. The line spectrum information was used to track the motion path of the speedboat, and to select the sliding window step distance ΔT=1 s according to the actual moving speed of the ship. The trajectory diagram drawn in MATLAB through the combined beamforming algorithm is shown in Figure 19. The duration of beamforming statistics was 50 s, and the angle of the ship changed by 20.95° relative to the hydrophone coordinates within 50 s, which is consistent with the track information recorded by GPS. The experimental results show that after attitude calibration and with the combined calculation analysis and processing, it is possible to obtain accurate DOA information from the noise radiated by the ship which was collected by the self-contained MEMS vector hydrophone.

## 5. Conclusions

In this paper, a self-contained MEMS vector hydrophone is designed and implemented, which can simultaneously collect the acoustic pressure and vibration velocity information in the sound field and realizes the self-contained storage of acoustic signals. Then, a combined acoustic pressure-vibration velocity beamforming algorithm was used for DOA estimation, which can effectively avoid the problem of ambiguity in the port and starboard. The indoor and outdoor experimental results show that the self-contained MEMS vector hydrophone has ideal low-frequency performance and DOA estimation capabilities. The research in this paper lays the foundation for the platform installation and engineering application of MEMS vector hydrophones in the future, and it is of great significance to marine low-frequency acoustic exploration. In order to further improve the spatial positioning capability of the hydrophone, future research will focus on the design of the 3D MEMS vector hydrophone.

## Figures and Tables

**Table 1:** Parameters of sensitive structure.

Structure Name	Parameter (µm)
Length of cantilever (L)	1000
Width of cantilever (b)	120
Thickness of cantilever (t)	40
Half side length of central block (a)	300
Radius of cilia (r)	200
Radius of outer cap wall (r2)	600
Height of cilia upper cylinder (h1)	1900
Height of hat cylinder (h2)	1000
Height of cilia lower cylinder (h3)	1500
Thickness of cap wall (d)	200
